# Colonic Wall Thickening Reported in Abdominal CT: Does It Always Imply Malignancy?

**DOI:** 10.1155/2019/2492097

**Published:** 2019-12-22

**Authors:** A. Akbas, H. Bakir, M. F. Dasiran, H. Dagmura, E. Daldal, Z. Ozsoy, Z. Ozmen, O. Demir, I. Okan

**Affiliations:** ^1^General Surgery and Surgical Oncology, Department of General Surgery, Bagcilar Training and Research Hospital, Istanbul 34100, Turkey; ^2^Department of General Surgery, Tokat Gaziosmanpasa University, Tokat 60100, Turkey; ^3^Department of Radiology, Tokat Gaziosmanpasa University, Tokat 60100, Turkey; ^4^Department of Biostatics, Tokat Gaziosmanpasa University, Tokat 60100, Turkey

## Abstract

**Aim/Background:**

Early diagnosis of patients with colon cancer is one of the most important parameters affecting the survival of patients. In this study, we aimed to examine the effect of the age, hemoglobin (Hb), albumin, neutrophil lymphocyte ratio (NLR), thrombocyte lymphocyte ratio (PLR), and mean platelet values (MPV) on the separation of benign and malignant diseases in patients with suspected colon wall thickness (CWT) observed in abdominal computed tomography (CT) examination.

**Method:**

The study included 116 patients between the ages of 18 and 95 who had CT examination where the colon wall could be evaluated and who also had colonoscopy. Patients suspected for CWT in CT with difficulties in differential diagnosis were divided into two groups according to colonoscopic-histopathological evaluations. Normal or benign pathological causes were included in the first group, while malignant causes constituted the second group. Whether the two groups differed in terms of CWT, Hb, age, albumin, NLR, PLR, and MPV values was investigated with descriptive statistics.

**Results:**

One hundred and sixteen patients (74 males, 42 females) with CT examinations and colonoscopic sampling were included in the study. After colonoscopic and histopathological evaluations, there were 64 cases in the first group and 52 in the second group. According to the results of the univariate analysis and multivariate logistic regression, CWT, Hb, NLR, and MPV were identified to be independent variables for determining colon cancer.

**Conclusion:**

A combined evaluation of Hb, NLR, and MPV values in patients with CWT in abdominal CT examination may contribute to the separation of benign and malignant pathologies.

## 1. Introduction

Computed tomography (CT) is a very common imaging method for the evaluation of abdominal pain [1]. Colon wall thickness increase (CWT) is a common finding nowadays [2]. CWT is a nonspecific finding and may not always indicate pathological condition. Benign (inflammatory causes, diverticulum, and polyps) and malignant (tumor) diseases could lead to CWT. Besides, causes such as solid-liquid stool, inadequate bowel distention, or contraction in the lumen could also be evaluated as CWT [3–5].

There is no consensus on the use of colonoscopy in patients with CWT based on CT results [5, 6]. Some advocate colonoscopy for these patients [7, 8] while some recommend colonoscopy only for the risky group [2, 9]. The American Gastroenterology Association still does not accept CWT as an absolute colonoscopy indication [1]. The current differences of opinion prevented the development of a common algorithm and left the decision to the clinic. Concerns about delay in diagnosis and legal pressure often lead clinicians to favor the use of colonoscopy, which is an invasive procedure [10]. Widespread use of colonoscopy may lead to increased health expenditure, prolonged appointment times, and some accompanying complications [11].

Our aim in this study was to evaluate the predictive effect of age, hemoglobin (Hb) and albumin levels, neutrophil lymphocyte ratio (NLR), platelet lymphocyte ratio (PLR), and mean platelet volume (MPV) in predicting the colonoscopy results in patients with CWT observed in CT. Early predicting of CWT due to malignant causes could reduce delays in diagnosis and treatment by performing colonoscopy in earlier stages.

## 2. Material Method

Ethics committee approval was received from Tokat Gaziosmanpaşa University Faculty of Medicine Ethics Committee. The phrase “colonic wall thickening” was scanned retrospectively through the University Health Research and Application Center/Hospital data processing database among the CT reports in which abdominal CT was taken and reported for any reason between January 1, 2013, and August 1, 2018. The files of these patients were examined one by one, and the ones that did not allow optimal measurement of the colon wall thickness in axillary CT sections (which did not comply with the extraction protocols of the abdominal CT taken with intravenous (IV) contrast material, which did not develop adequate distension in the colon, which included solid-liquid stool in the colon); the cases who had heart failure, hypoalbuminemia, and nephrotic syndrome that could affect colon wall thickness; the cases who underwent surgery and who were monitored for known bowel disease or anemia; the cases with CT findings strongly suggesting CWT; the cases who were diagnosed to have CWT with CT but who did not have colonoscopy in our hospital; or whose colonoscopy data were not of sufficient quality were excluded from the study. The patients who underwent appropriate oral+IV contrast CT imaging protocols, for whom optimal colon wall thickness could be measured; who underwent colonoscopy and blood tests in our hospital (within one month after CT scan); and who were evaluated by biopsy were included in the study. Demographic data, Hb, albumin, NLR, PLR, and MPV values of the cases were obtained from electronic files. The abdominal CT sections of the patients were reevaluated by two experienced radiologists unaware of the results of colonoscopic-histopathological evaluation. CWT greater than five millimeters (mm) was considered pathological. Patients who were suspected to have CWT in CT and who were difficult in differential diagnosis were divided into two groups according to colonoscopic-histopathological evaluation results. Patients with normal colonoscopic findings and for whom no further examination were required, as well as cases with wall thickness due to benign causes (inflammatory causes, diverticulum, and polyps), were included in the first group, and the patients found to be malignant (tumor) in pathological evaluation were included in the second group. Whether the two groups differed in terms of CWT, Hb, age, albumin, NLR, PLR, and MPV values was investigated with descriptive statistics.

Descriptive analyses were conducted to give information about the general characteristics of the study groups. Data of continuous variables were expressed as mean ± standard deviation; categorical variables were given as *n* (%). When comparing the averages of the quantitative variables between the groups, the significance test of the difference between two means and one-way analysis of variance were used. For variables found to be significantly different between the groups based on one-way analysis of variance, Tukey's HSD test was used for multiple comparisons. Cross-tables were created for the qualitative variables and chi-squared tests were used for the relationships between the related variables. *p* < 0.05 was considered statistically significant. Receiver operating characteristic (ROC) curve analysis was used to identify significant parameters in multivariate analysis. Calculations were made using SPSS statistics software (IBM SPSS Statistics ver. 19, SPSS Inc., an IBM Co., Somers, NY).

## 3. Results

Colon wall thickness increase was detected in abdominal CT reports of 179 patients. Sixty-three patients who did not meet the study criteria were excluded. One hundred and sixteen patients (74 males, 42 females) with CT examinations and colonoscopic sampling (average age: 63.50 ± 13.85) were included in the study. In the evaluation, there were 64 cases in the first group and 52 cases in the second group (Table 1).

In the univariate analysis between group I and group II, there was a significant difference between CWT, Hb, albumin, NLR, PLR, and MPV (*p* < 0.001), but there was no difference in age (*p* = 0.27). In addition, multivariate logistic regression analysis between group I and group II showed that CWT, Hb, NLR, and MPV were independent variables in the detection of colon cancer (Table 2).

**Figure 1:**
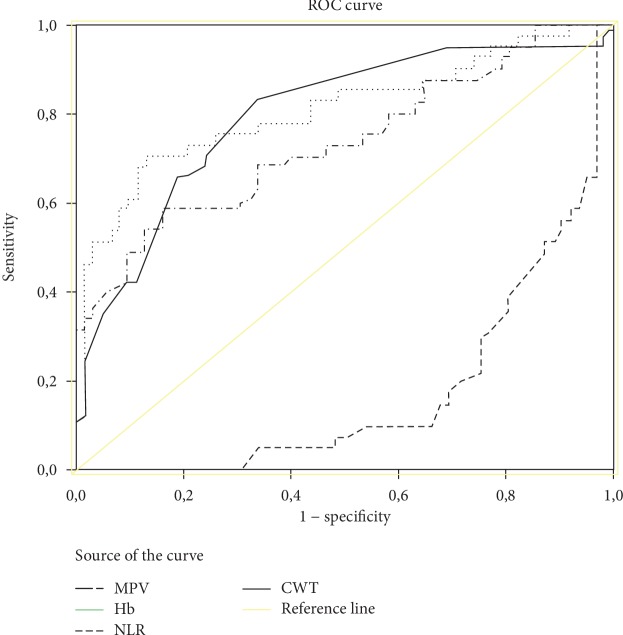
Receiver operating characteristic (ROC) curve analyses of significant parameters for the diagnosis of colon cancer. CWT: colon wall thickness; Hb: hemoglobin; MPV: mean platelet volume; NLR: neutrophil-to-lymphocyte ratio.

In ROC curve analyses of these independent variables, AUC was above 0.600 for CWT, Hb, NLR, and MPV (Figure 1). Proposed cutoff values and performance characteristics for these variables are shown in Table 2.

## 4. Discussion

Acute and chronic abdominal pain is an important cause for emergency room visits. CT has been an increasingly used method for the assessment of abdominal pain [5, 12]. CWT has become a common finding as a result of the widespread use of CT [13]. Conventional CT images have low specificity and sensitivity to CWT [14]. This finding may be a variant of normal or may be due to benign or malignant diseases. For this reason, advanced endoscopic evaluations such as colonoscopy are needed to determine the etiology that may cause CWT [5, 15].

There are many studies in the literature evaluating CWT observed in CT. The results and recommendations of these studies are mixed [2, 6]. Wolff et al. [6], in their study, identified 7.4% malignant, 66.3% benign, and 26.1% normal findings in patients, while Eskaros et al. [16] reported 64% pathological findings. Similarly, Kathawala and Cooper [17] evaluated 60 patients with WT on CT by colonoscopy and identified tumors in 9%. Based on these results, Wolff et al. [6], Eskaros et al. [16], and Kathawala and Cooper [17] recommended colonoscopy for all patients with CWT on CT. However, there are studies in the literature suggesting that colonoscopy should be performed only when there is a risk factor in patients with CWT. Khan et al. [2], in their study, identified cancer in 5.7% of patients while 65.7% of patients had wall thickening due to benign causes. Stermer et al. [18] et al. found no malignant findings in their study that reported CWT in 34.7% of the patients due to benign causes. Khan et al. [2] and Stermer [18] suggested colonoscopy for the risky groups which include patients with anemia, patients over fifty years of age, and patients whose fecal occult blood test was positive. In our study, 31.9% of patients were observed to have CWT due to benign causes and 44.8% were observed to have CWT secondary to malignancy. The fact that our hospital is a tertiary treatment center and that the majority of our cases were referred patients could have affected the average age and percentage of patients with malignancy.

Distension of the colonic wall is very important for normal colonic wall thickness measurement. When the colon is inflated, the wall thickness is less than 3 mm [19]. In CT taken under optimal conditions, the thickness of the colonic wall is considered normal up to 3 mm and pathological above 5 mm [8]. Colonic redundancy with solid-liquid stool makes it difficult to measure wall thickness. Contrast involvement and wall thickness are important in the differential diagnosis of CWT. CWT due to malignant causes is generally more than 20 mm and with homogeneous contrast enhancement. In CT under optimal conditions, there is not much confusion in the diagnosis of CWT over 20 mm. Moderate CWT (<20 mm) is a more complex condition and is most often caused by benign events (inflammatory causes, diverticulum, and polyps). To a lesser extent, it can also be seen due to overlapping and malignant reasons arising from the colon wall [20]. In our study, CWT was 9.43 ± 3.89 mm in group I patients and 16.21 ± 10.3 mm in group II patients. Univariate analysis revealed statistical difference between the two groups (*p* < 0.001). In addition, multivariate logistic regression analysis showed that CWT was an independent risk factor for colon cancer (OR: 1.182; 95% CI: 1.036-1.348; *p* = 0.013) (Table 2) (ROC analysis cutoff value for CWT > 9 mm: AUC: 0.800; 95% CI: 0.718-0.871; sensitivity 82%; specificity 0.65%; *p* < 0.001) (Table 3, Figure 1). In the evaluation of patients with suspected CWT in CT, a holistic evaluation of other parameters that may help to distinguish between malignant and benign diseases may contribute to the clinician's decision (Tables 2 and 3).

The most common cause of iron deficiency anemia in premenopausal women is menstrual loss. In men and postmenopausal women, the most common cause is gastrointestinal losses. Gastrointestinal system cancers may occur with iron deficiency anemia. Exclusion of these diseases in the presence of anemia has clinical importance and priority. Anemia is seen in 11-55% of cases with colon cancer [21–23]. In a study conducted on men over 60 years of age in the UK, in the event of Hb < 11 g/dl and iron deficiency anemia, colon cancer incidence rate was 13.3% while in women of the same age group with Hb < 10 g/dl, this rate was reported to be 7.7% [23]. Similarly, there are numerous studies reporting that albumin value is lower than normal in malignancies originating from the gastrointestinal tract [24, 25]. In univariate analyses in the present study, Hb and albumin levels were significantly different between group I and group II patients (*p* < 0.001), whereas multivariate logistic regression results indicated Hb as independent variable (OR: 0.566; 95% CI: 0.350-0.916; *p* = 0.021) (Table 2) (cutoff value in ROC analysis for Hb ≤ 12.4 g/dl: AUC: 0.800; 95% CI: 0.716-0.869; sensitivity 86%; specificity 0.65%; *p* < 0.001) (Table 3, Figure 1). Hb and albumin levels secondary to malnutrition in malignant patients are expected to be low. Inclusion of these parameters in the evaluation of patients with suspected (moderate) CWT may contribute to the differential diagnosis [23, 25]. Lack of significant differences between the study groups for the albumin level in the present study could be due to the fact that the cases considered to have malignancy based on CT findings were excluded from the study.

It is known that gastrointestinal system cancers occur at an advanced age and increase with age [25, 26]. There was no statistically significant difference between benign and malignant groups due to the high mean age of the patient groups (*p* = 0.27) (Table 2).

Inflammation in tumor cells increases angiogenesis and is effective in the development and progression of many cancers [27]. Neutrophils, lymphocytes, and platelets play an important role in tumor inflammation and immunology [28]. The hemopoietic response of inflammatory markers (platelets, lymphocytes, neutrophils) in the blood due to cytokines released by tumor cells results in an increase in the number of neutrophils and platelets and a decrease in the number of lymphocytes [29, 30]. Since the physiological response of circulating leukocytes to stress causes an increase in neutrophil count and a decrease in lymphocyte count, the ratio of these two subgroups to each other (NLR) is used as an indicator of inflammation [30, 31]. The presence of T lymphocytes in tumor tissue is indicative of a marked immune response to the lesion. Studies have shown that the low number of lymphocytes in colorectal cancers is an indicator of poor prognosis [32, 33]. There are studies indicating that NLR is a simple method that can be used to determine the poor prognosis in patients with colorectal cancer in the preoperative period [34–36]. Oflazoglu et al. [37], in their study on 338 colorectal cancer patients, mentioned that NLR could be used as a marker in patients with colorectal cancer. In the univariate analysis of our study, NLR was significantly different between group I and group II (*p* < 0.001) and multivariate logistic regression results showed that NLR was an independent variable in predicting colon cancer (OR: 1.944; 95% CI: 0.991-3.813; *p* = 0.043) (Table 2) (cutoff value in ROC analysis for NLR > 3.06: AUC: 0.840; 95% CI: 0.760-0.901; sensitivity 75%; specificity 0.87%, *p* < 0.001) (Table 3, Figure 1).

Platelets, on the other hand, play an important and multifaceted role in cancer progression. Platelets can increase angiogenesis and stimulate tumor growth by cytokines (interleukin-6) and vascular endothelial growth factor [38, 39]. In the study by Karagöz et al. [40] and Pedersen and Milman [41], MPV and platelet count were significantly higher in lung cancer patients compared to the normal group. Oflazoglu et al. [37] stated that PLR and MPV increased in patients with colorectal cancer and that it could be used as a reliable prognostic marker. Anuk et al. [39] compared the cases that they operated for ileus into two groups as benign and malignant according to etiologic causes and compared the rates of PLT and MPV. Both values were higher in malignant group. Ma et al. [42] compared normal patients with epithelial ovarian tumor patients and found significantly higher MPV and PLR in patients with tumor. In the present study, PLR and MPV were statistically different between group I and group II (*p* < 0.001). In addition, multivariate logistic regression results showed that PLR was not significantly different between the two groups, whereas MPV was an independent variable in determining colon cancer (OR: 1.851; 95% CI: 1.13-3.032; *p* = 0.014) (Table 2; cutoff value in ROC analysis for MPV > 8.09 fL: AUC: 0.732; 95% CI: 0.641-0.811; sensitivity 57%; specificity 0.83%; *p* < 0.001) (Table 3, Figure 1).

**Figure 2:**
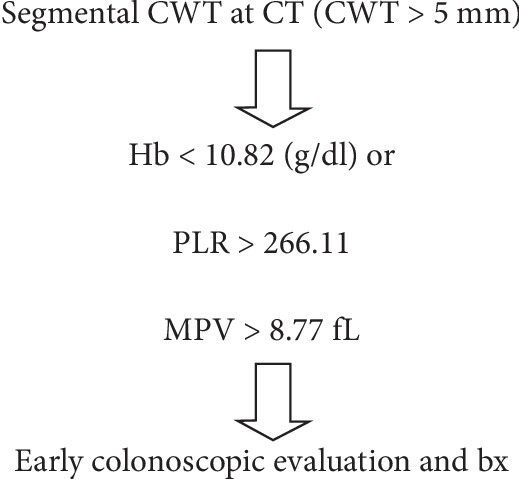
Management tool for colon wall thickening (CWT) to neoplasia with high positive predictive value.

The presence of suspected (moderate) CWT in CT is often considered to be benign or a variant of normal but may also be due to malignant causes. When in doubt, clinicians often decide to perform a colonoscopy. Colonoscopy is an invasive procedure and may lead to complications, loss of labor, and increased health expenditures [11]. Easily accessible and cost-effective parameters that could contribute to the decision-making of the clinician could provide great convenience in the differential diagnosis and decrease the health expenditures, lowering the undesirable consequences such as labor loss and complications. Hb, albumin, NLR, PLR, and MPV are robust and inexpensive parameters that can easily be detected in any hospital. We believe that the evaluation of Hb, age, albumin, PLR, NLR, and MPV parameters with larger prospective cohort studies and, if possible, the development of an algorithm in this regard could be useful in differential diagnosis of patients with suspected CWT (Figure 2).

Our study carries the inherent drawbacks of retrospective studies and has some limitations. Among them are inclusion of only the patients with suspected (moderate) CWT who had colonoscopy in our hospital and limited number of patients, both of which may have affected the results.

## 5. Conclusion

Hb, NLR, and MPV differed significantly between benign and malignant CWT cases. Evaluation of patients with suspected (moderate) CWT on CT using Hb, NLR, and MPV may contribute to the diagnosis. Further studies could be useful to validate our results.

## Figures and Tables

**Table 1:** Demographic data and results of colonoscopy and histopathological evaluation of the patients.

		Group I	Group II	Total (*n*) (%)
Female		23	19	42 (36.2%)
Male		41	33	74 (63.8%)
Age (years)		62.23 ± 14.72	65.08 ± 12.67	
Colonoscopic-histopathological evaluation		(*n*)	(*n*)	
Colonoscopy with no finding		27	—	27 (23.2%)
Benign	Polyps	22	—	37 (31.9%)
	Colitis	10	—	
	Diverticulum	5	—	
Malign	Adenocarcinoma	—	51	52 (44.8%)
	Lymphoma	—	1	
Total (*n*)		64	52	116

**Table 2:** Comparison of the two groups.

	Univariate analysis			Multivariate analysis		
	Group I	Group II	*p*	OR	95% CI (min–max)	*p*
Number (*n*)	64	52				
Age (years)	62.23 ± 14.72	65.08 ± 12.67	0.27	0.993	0.948-1.041	0.778
Albumin (g/dl)	3.95 ± 0.76	3.41 ± 0.7	<0.001	0.963	0.361-2.57	0.940
CWT (mm)	9.43 ± 3.89	16.21 ± 10.3	<0.001	1.182	1.036-1.348	0.013
MPV (fL)	7.2 ± 1.14	8.77 ± 1.97	<0.001	1.851	1.13-3.032	0.014
Hb (g/dl)	12.99 ± 1.93	10.82 ± 1.7	<0.001	0.566	0.35-0.916	0.021
NLR	2.11 ± 1.13	5.49 ± 3.7	<0.001	1.944	0.991-3.813	0.043
PLR	119.88 ± 65.34	266.11 ± 202.76	<0.001	0.996	0.983-1.01	0.608

OR: odds ratio; CWT: colon wall thickness; Hb: hemoglobin; MPV: mean platelet volume; NLR: neutrophil-to-lymphocyte ratio; PLR: platelet-to-lymphocyte ratio.

**Table 3:** The results of ROC analysis.

Variable	Cutoff value	AUC	95% CI	Sensitivity	Specificity	*p*
CWT	>9	0.802	0.718-0.871	0.827	0.656	<0.001
Hb (g/dl)	≤12.4	0.800	0.716-0.869	0.865	0.656	<0.001
NLR	>3.06	0.840	0.760-0.901	0.750	0.875	<0.001
MPV (fL)	>8.09	0.732	0.641-0.811	0.577	0.839	<0.001

AUC: area under the curve; OR: odds ratio; CWT: colon wall thickness; Hb: hemoglobin; MPV: mean platelet volume; NLR: neutrophil-to-lymphocyte ratio.

## Data Availability

A brief description of the ethical or legal restrictions on the dataset. A contact to whom requests for the data may be sent.
